# A Homozygous *IER3IP1* Mutation Causes Microcephaly With Simplified Gyral Pattern, Epilepsy, and Permanent Neonatal Diabetes Syndrome (MEDS)

**DOI:** 10.1002/ajmg.a.35583

**Published:** 2012-09-18

**Authors:** Ghada MH Abdel-Salam, Ashleigh E Schaffer, Maha S Zaki, Tracy Dixon-Salazar, Inas S Mostafa, Hanan H Afifi, Joseph G Gleeson

**Affiliations:** 1 Human Genetics and Genome Research Division, Clinical Genetics Department, National Research CentreCairo, Egypt; 2 Department of Neuroscience, Howard Hughes Medical Institute, University of CaliforniaSan Diego, La Jolla, California; 3 Human Genetics and Genome Research Division, Orodental Genetics Department, National Research CentreCairo, Egypt

**Keywords:** microcephaly, epilepsy, autosomal recessive, infantile diabetes mellitus, burst suppression, Wolcott–Rallison syndrome

## Abstract

Wolcott–Rallison syndrome (WRS) and the recently delineated microcephaly with simplified gyration, epilepsy, and permanent neonatal diabetes syndrome (MEDS) are clinically overlapping autosomal recessive disorders characterized by early onset diabetes, skeletal defects, and growth retardation. While liver and renal symptoms are more severe in WRS, neurodevelopmental characteristics are more pronounced in MEDS patients, in which microcephaly and uncontrolled epilepsy are uniformly present. Mutations in the *EIF2AK3* gene were described in patients with WRS and defects in this gene lead to increased susceptibility to apoptotic cell death. Mutations in *IER3IP1* have been reported in patients with MEDS and similarly, loss of activity results in apoptosis of neurons and pancreatic beta cells in patients. Here we report on a homozygous mutation of the *IER3IP1* gene in four patients from two unrelated consanguineous Egyptian families presenting with MEDS who display burst suppression patterns on EEG. All patients presented with mildly elevated liver enzymes, microalbuminuria, and skeletal changes such as scoliosis and osteopenia, leading to repeated bone fractures. We expand the phenotypic spectrum of MEDS caused by *IER3IP1* gene mutations and propose that WRS and MEDS are overlapping clinical syndromes, displaying significant gene-dependent clinical variability. © 2012 Wiley Periodicals, Inc.

## INTRODUCTION

Microcephaly with simplified gyration is defined as congenital microcephaly associated with a reduced number of gyri, thin or normal cortical thickness, and reduced number and depth of sulci. It may be associated with abnormal myelination or additional brain malformations, and it is classified into five groups according to associated brain imaging findings [Basel-Vanagaite and Dobyns, 2010]. Congenital microcephaly is estimated to affect 1.3–150 per 100,000 live births [Kaindl et al., 2010]. Infantile-onset diabetes mellitus is also a rare disorder, which may be either transient or permanent. The permanent form accounts for approximately 50% of cases and has an estimated incidence of 1 in 260,000 live births [Stanik et al., 2007; Slingerland et al., 2009]. Permanent neonatal diabetes can occur isolated, or accompanied by neurological features such as cerebellar hypoplasia (*PTF1A* mutations) [Sellick et al., 2004], congenital hypothyroidism (*GLIS3* mutations) [Senee et al., 2006], developmental delay (*KCNJ11* mutations), and epilepsy (DEND syndrome) [Gloyn et al., 2006].

Wolcott–Rallison syndrome (WRS, OMIM#226980) is a rare autosomal recessive condition characterized by permanent neonatal diabetes, epiphyseal dysplasia, hepatic dysfunction, and renal impairment and is caused by mutations in the *EIF2AK3* gene [Delepine et al., 2000]. *EIF2AK3* is one of four kinases known to phosphorylate *EIF2A*, a translation initiation factor crucial for protein synthesis, and mutations in this gene are thought to cause Wolcott–Rallison syndrome by increasing endoplasmic reticulum stress and inducing subsequent apoptotic cell death [Delepine et al., 2000; Biason-Lauber et al., 2002; Jiang et al., 2003]. Recently, Poulton et al. [2011] described two unrelated consanguineous families with an overlapping but unique clinical syndrome displaying microcephaly, simplified gyral pattern, severe infantile epileptic encephalopathy, and permanent neonatal diabetes mellitus (MED syndrome or MEDS, OMIM#614231). Molecular analysis in patients with MEDS revealed homozygous missense mutations in the immediate early response three interacting protein 1 gene (*IER3IP1*), an ER stress response protein that mediates cell differentiation [Poulton et al., 2011]. These patients had elevated apoptosis of the cerebral cortex and pancreatic beta cells, which was hypothesized to be the result of dysfunctional cellular differentiation under “stress” stimuli [Poulton et al., 2011].

Here, we report on the clinical, radiological, and molecular findings of four patients with MEDS and *IER3IP1* mutations who display burst suppression on EEG, elevated liver enzymes, and microalbuminuria. Our work provides independent confirmation that mutations in *IER2IP1* underlie MEDS and expands the phenotypic spectrum of this syndrome. We propose MEDS and Wolcott–Rallison syndrome as overlapping clinical syndromes that display significant gene-dependent clinical variability.

### Patient Data

#### Family MEDS-1251, Patient 1

**FIG. 1:**
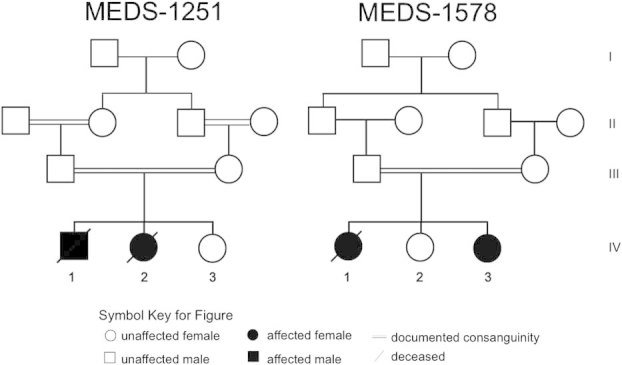
The pedigree of the families included in the study.

**FIG. 2:**
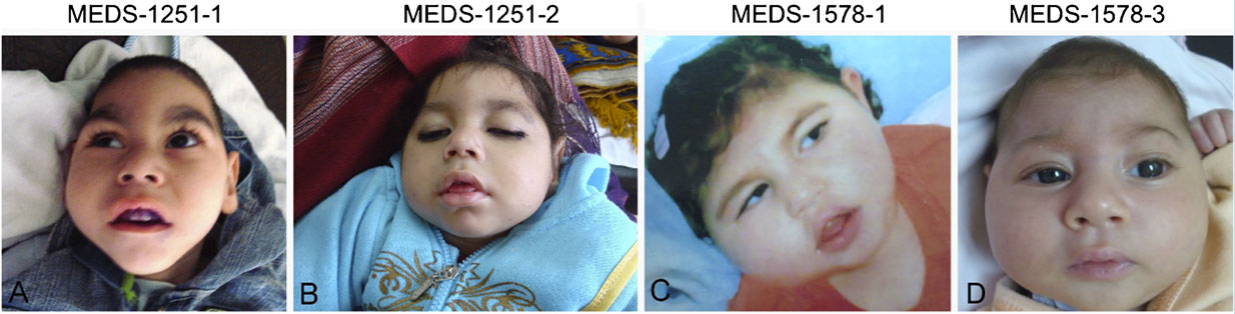
The facial features of Patients. Note narrow/short forehead with bitemporal grooving, anteverted nares, deep philtrum, and tented vermilion of upper lip.

This male patient was the first child born to first-cousin healthy Egyptian parents (Fig. 1), mother age 23 years old and father age 32 years at the time of delivery. They had a family history of type 2 diabetes mellitus in maternal grandmothers, but were otherwise unremarkable. The pregnancy was uneventful and he was delivered at term vaginally, with weight approximately 3,000 g. Birth length, head circumference, and Apgar scores were not recorded. He had a weak cry and was lethargic but there were no other immediate postnatal problems. He fed well and was discharged within 24 hr of birth. He was internalized due to high fever and was diagnosed as having bronchopneumonia. Persistent hyperglycemia was detected and he was diagnosed with permanent neonatal diabetes. Further, tonic–clonic hemiconvulsions were noticed, showing poor response to sodium valproate and diazepam. A brain computed tomography scan at this age showed acute hematoma in the falx cerebri and subependymal hemorrhage and the electroencephalogram (EEG) showed polyspikes and slow waves with burst suppression pattern. He continued to have repeated pneumonias and the chest radiography showed prominent pulmonary vasculature of lungs bilaterally. Myoclonic and generalized tonic–clonic seizures were noticed, and were intractable to anti-epileptic drug therapy. Urinary amino acids and organic acid screens were unremarkable. Serum lactate was mildly elevated (3.1 mmol/L with reference range 0.5–2 mmol/L). The child showed almost no neurodevelopmental progress. He was spoon fed initially but required intervention via gastrostomy tube at the age of 3 years. Optic fundus examination was normal. Family was referred to our clinics when he was 2½ years of age for genetic counseling since they had a second child with similar clinical findings. On physical examination, weight was 7,200 g (−4 SD), length was 81 cm (−2.5 SD), and head circumference was 37.8 cm (−8 SD). The child had short forehead with bitemporal narrowing, anteverted nares, deep philtrum, and tented vermilion of upper lip (Fig. 2A). Bilateral clinodactyly, limitation of flexion at the knee joint, scoliosis, and puffiness at the dorsum of feet were noticed. The patient had hirsutism, bilateral undescended testes, and hypoplastic scrotum. He showed truncal hypotonia and did not appear to respond to visual or auditory stimuli. He was severely disabled and died at the age of 5½ resulting from pneumonia. Autopsy was denied.

**FIG. 3:**
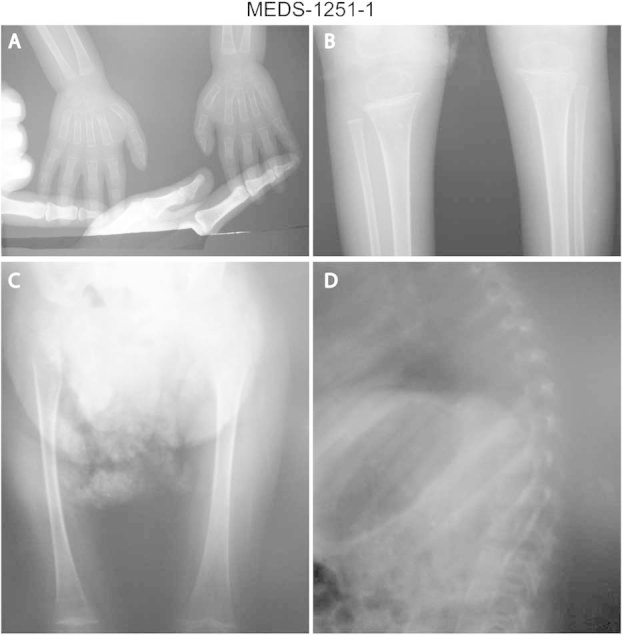
Radiograph of Patient 1. **A**: Osteopenia in carpal, metacarpal and phalanges with cone shaped proximal phalanges. **B**: Thin cortex of tibia and fibula with metaphyseal widening and osteopenia. **C**: Thin cortex of femora with severe osteopenia. **D**: Kyphosis, severe osteopenia with platyspondyly and osteopenic ribs.

**FIG. 4:**
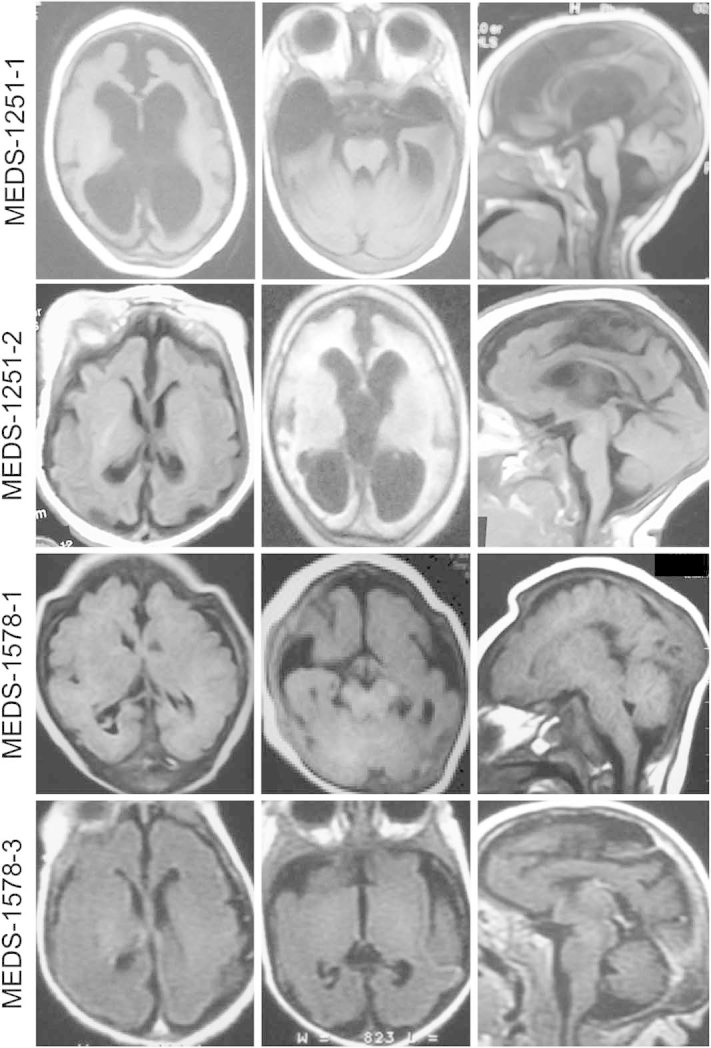
Cranial MRI of patients. **First column**: Rostral axial T1 weighted images show simplified gyral pattern, severe microcephaly, and different degree of ventriculomegaly, suggestive for cerebral atrophy. **Second column**: Caudal axial T1 weighted images showing brain atrophy and simplified gyral pattern. **Third column**: Sagittal images showing severely reduced volume of corpus callosum and cerebellar vermis hypoplasia.

His medical history revealed that he had two pathological fractures of left femur at 2 and 4 years of age. Radiography revealed osteoporosis, cortical thinning of long bones, and metaphyseal changes (flaring; Fig. 3A–D). Hematological indices showed microcytic hypochromic anemia and thrombocytopenia (platelets 7.8 × 10^4^), and liver enzymes were mildly elevated on different occasions. Echocardiography and abdominal sonography were normal. There was no evidence of pancreatic or renal anomalies; however, urine analysis consistently showed microalbuminuria. G-banded karyotype at 550–600 band levels revealed normal male. Brain magnetic resonance imaging (MRI; Fig. 4) revealed microcephaly with simplified gyration, cortical atrophy, hypoplastic corpus callosum, and cerebellar vermis hypoplasia.

#### Family MEDS-1251, Patient 2

Patient 2 is the younger sister of Patient 1 (Fig. 1) and was born at term by elective cesarean weighing 3,540 g (75th centile). Head circumference, length and Apgar score were not recorded. The neonatal period was uneventful except for physiological jaundice. At the age of 2 months, little developmental progress was noted; she was barely able to raise her head. Hypomotor seizures with abnormal eye deviation were noticed after the first shot of DPT vaccine. EEG showed generalized epileptic abnormalities with sharp and slow-waves. She had marked hyperglycemia that was treated with continuous insulin infusion. Her seizures and hyperglycemia were difficult to control. She had recurrent respiratory tract infections, feeding difficulties and she spent most of her first year of life in the hospital. During this time, she had generalized hypotonia and made almost no developmental progress: she was unable to roll or sit, there were no vocalizations beyond an infrequent moaning, and required a gastrostomy tube for feeding. At the age of 18 months, her weight, length, and head circumference were 5,500 g (−5 SD), 70.5 cm (−3.3 SD), and 34 cm (−9 SD), respectively. She had similar facial features like her sibling (Fig. 2B). She had profound intellectual disability, truncal hypotonia with decreased muscular bulk, and increased lower extremity deep tendon reflexes. She was unable to follow objects visually, and to independently sit. At 18 months, seizures changed to myoclonic, which were intractable to multidrug therapy. EEG showed burst suppression pattern. Ophthalmological examination showed neither retinal nor optic disc abnormalities. Cranial MRI showed simplified gyral pattern and agenesis of corpus callosum with mild cerebellar vermis hypoplasia without atrophy (Fig. 4). She experienced a pathological fracture of the proximal right tibia at the age of 20 months that took several months to heal. At 26 months of age she developed bronchopneumonia and died. Autopsy was declined by the parents.

#### Family MEDS-1578, Patient 1

The firstborn child of healthy first cousin 27-year-old mother and 33-year-old father was born at term by vaginal delivery after an uncomplicated pregnancy with a weight of 2,700 g (Fig. 1). There was a strong family history of type 2 diabetes mellitus in the grandparents and their siblings. Head circumference, length, and Apgar scores were not recorded. Abnormal jerking movements, thought to be seizures, began few hours after birth, and continued despite multiple anticonvulsants. At 40 days of age she suffered from a respiratory tract infection with high fever and severe vomiting. She became progressively lethargic and required admission to the hospital. Routine biochemical evaluation showed hyperglycemia and ketone bodies in the urine. Treatment with insulin was initiated; however, her blood glucose levels were difficult to control. At 2 months of age, weight, length, and head circumference were approximately 4,300 g, 55 cm (mean), and 31 cm (−3.6 SD), respectively. She had bitemporal narrowing, puffy cheeks, anteverted nares, short/narrow forehead, tented vermilion of upper lip, high narrow palate, gingival hypertrophy, open-mouth, and bilateral 5th finger clinodactyly (Fig. 2C). Generalized edema developed over the first few months of life, most marked over the distal limbs. Axial hypotonia with brisk tendon reflexes were noted. Daily myoclonic seizures were refractory to multiple antiepileptic drugs. She made almost no developmental progress. There were no vocalizations except for occasional groaning. She had poor visual responsiveness and ophthalmologic examination showed optic atrophy. There was no history of long bone fractures. Abdominal ultrasound and CT scan, and echocardiography were normal. Results of plasma urea, electrolyte, and creatinine levels, liver function tests, urine and serum amino acids and organic acids, very long chain fatty acids, serum lactate, and ammonia were normal. Microalbuminuria was a constant finding observed in urine analysis. Chromosome analysis on lymphocytes at 550–600 band levels revealed normal female karyotype. The EEG at 8 weeks of age showed burst suppression pattern. Cranial MRI showed cerebral atrophy with simplified gyral pattern, and agenesis of the corpus callosum, but no cerebellar atrophy (Fig. 4). Radiograph showed poor modeling of long bones and osteopenia. She died at the age of 3½ years. Autopsy was not performed.

#### Family MEDS-1578, Patient 2

The third child, and second affected, in this family (Fig. 1) had fetal ultrasonography at 32 weeks of gestation showing small biparietal diameter. She was born after an uneventful pregnancy by cesarean at 37 weeks of gestation with a birth weight of 2,300 g (−3 SD). Parents were told that the birth head circumference was small but it was not recorded. At birth she had neonatal jaundice, but blood glucose levels were within normal range. Two weeks later, she was found to have hyperglycemia and was diagnosed with neonatal diabetes. At the age of 50 days, weight, length, and head circumference were 4,000 g (mean), 54 cm (mean), and 29.5 cm (−4.6 SD), respectively. Bitemporal narrowing, puffy cheeks, anteverted nares, tented vermilion of upper lip, short/narrow forehead, high narrow palate, and gingival hypertrophy were noticed (Fig. 2D). She had normal grasp and Moro reflexes. Normal tone and brisk reflexes were elicited. There was social smile and visual fixation. Shortly after examination, she developed hemiconvulsions with occasional eye and mouth twitches. Myoclonic jerks were also observed. EEG recordings of these episodes revealed burst suppression pattern. Seizures partially responded to phenobarbital and topiramate. Cranial MRI showed simplified gyral pattern and agenesis of corpus callosum with normal cerebellum and brain stem (Fig. 4).

## METHODS

This study was approved by the Institutional Review Board at the University of California, San Diego and collaborating institutions, all able study participants signed informed consent documents, and the study was performed in accordance with HIPPA privacy rules. DNA was extracted from blood using Qiagen reagents. SNP genotyping was performed using the Sequenom MassARRAY system and iPLEX reagents according to manufacturers recommendations. DNA was subjected to exome capture with the Agilent SureSelect Human All Exome 50 Mb kit, sequenced on an Illumina HiSeq2000 instrument, resulting in ∼94% target coverage at >10× depth. BWA was utilized to align the sequences to the human reference genome (hg19), and GATK [McKenna et al., 2010] was used for variant identification.

## RESULTS

### Molecular Analysis

**FIG. 5:**
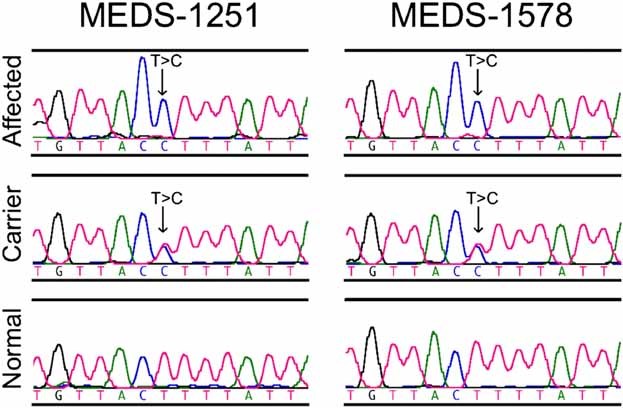
Sequence chromatograms of exon 3 of the IER3IP1 gene showing the point mutation c.T233C detected as homozygous in the affected patients from each family, heterozygous in both parents, and homozygous wildtype in both unaffected siblings.

Due to the recent report of occasional microcephaly and NPD resulting from mutations within the *EIF2AK3* gene [de Wit et al., 2006], we analyzed markers rs4832163 and rs735738, both of which were recombinant in the family, which excluded the locus as causative in this family. We similarly excluded the genes and loci *MCPH 1-7*, known to cause primary microcephaly. We then performed whole exome sequencing [Park et al., 2008] on the younger affected child from both families. The identified variants from WES were prioritized based upon their allele frequency in our internal patient database, amino acid conservation, and expression data from the GEO profile. Of these variants, a previously described causative homozygous mutation (c.T233C) in *IER3IP1* was identified in both families [Poulton et al., 2011]. This variant occurs in exon 3 of *IER3IP1* and converts a leucine to a proline at amino acid position 78. This homozygous variant was confirmed to be present in all affected children, while the healthy siblings were both homozygous for the wildtype allele (Fig. 5).

## DISCUSSION

In 2005, de Wit et al. described three patients with microcephaly with simplified gyration and skeletal deformities with *EIF2AK3* mutations, pointing to the diagnosis of Wolcott–Rallison syndrome in two of the three patients reported [de Wit et al., 2006]. Subsequently, the same group reported homozygous missense *IER3IP1* mutations in the previously described patient negative for mutations in *EIF2AK3*, as well as in an additional unrelated family, concluding the diagnosis of MEDS [Poulton et al., 2011]. The patients with *IER3IP1* mutations presented with more severe microcephaly, permanent neonatal diabetes, and skeletal deformities as compared with patients harboring *EIF2AK3* mutations. Furthermore, uncontrolled seizures and burst suppression EEG pattern were noticed in all patients with *IER3IP1* mutations. Here we describe four patients from two unrelated families showing clinical features of MEDS with burst suppression EEG pattern, skeletal deformity consisting of scoliosis and cortical thinning of long bones leading to multiple fractures, slightly elevated liver enzymes, and microalbuminuria. This report expands the phenotype of MEDS caused by *IER3IP1* gene mutations. We therefore conclude that Wolcott–Rallison syndrome and MEDS are clinically overlapping syndromes that display gene-dependent clinical variability.

The patients reported here had many overlapping features with Wolcott–Rallison syndrome (Table I). All the patients reported here had permanent neonatal diabetes as described in Wolcott–Rallison syndrome. However, the age of onset for diabetes in Wolcott–Rallison syndrome is variable, ranging from 2 weeks [Wolcott and Rallison, 1972] to 30 months in some cases [Castelnau et al., 2000; Iyer et al., 2004; Ozbek et al., 2010]; whereas our patients display infantile onset permanent neonatal diabetes. Similar to Wolcott–Rallison syndrome, our patients displayed growth deficiency and skeletal changes, including irregular epiphyses, flat acetabulum, enlarged metaphyses, scoliosis, occasional kyphosis, and profound osteopenia with increased fracture susceptibility of the long bones [Ozbek et al., 2010; Senee et al., 2006]. In addition, Patients 1 and 2 (Family MEDS-1251) also showed very severely retarded bone age, suggestive that this feature may be unique for patients with *IER3IP1* mutations. Similar to what was reported by Poulton et al. [2011], elevated liver enzymes leading to terminal hepatic failure predominant in Wolcott–Rallison syndrome, were absent in our patients. Moreover, in Wolcott–Rallison syndrome, renal dysfunction, with persistent proteinuria leading to renal failure, was reported in 45% of the patients [Ozbek et al., 2010]. Although, all of our patients had microalbuminuria, none developed renal failure. Additionally, nearly 62% of the patients with Wolcott–Rallison syndrome showed mild to moderate developmental delay and mental retardation [Ozbek et al., 2010] while only two Wolcott–Rallison syndrome patients (6%) had microcephaly with simplified gyration, and cerebellar hypoplasia. In contrast, all of our patients with MEDS display microcephaly, simplified gyral pattern, and hypoplasia of the corpus collosum; demonstrating the rarity of these features in Wolcott–Rallison syndrome [Iyer et al., 2004], and its common occurrence in MEDS. Moreover, our patients displayed profound mental retardation.

**TABLE I:** Clinical Features of Present Patients Compared With PEHO, Wolcott–Rallison and MEDS Syndrome

Variables	PEHO syndrome [Riikonen et al., 1999; Somer, 1993]	Wolcott–Rallison syndrome [Iyer et al., 2004]	MEDS [Poulton et al., 2011]	Present report
Microcephaly	At birth	Not all patients had congenital microcephaly	2/2 Congenital microcephaly	4/4 Congenital microcephaly
Neonatal hypotonia	Infantile	Not reported	2/2	4/4
Early onset insulin-dependent diabetes mellitus	Not reported	All patients	1/2 reported	4/4
Insulin/IGF	Low	Not reported	Not reported	Not performed
Type of seizures	Myoclonic seizures and infantile spasms	Uncommon manifestations, generalized seizures	2/2 Myoclonic and tonic–clonic	4/4 Myoclonic seizures and infantile spasms
EEG pattern	Hypsarrhythmia	Non-specific	2/2 Hypsarrhythmia	4/4 Burst suppression
Response to anti epileptic drugs	Poor	Poor	2/2 Poor	4/4 Poor
Psychomotor retardation	Profound	Severe	2/2 Severe	4/4 Profound
Brain imaging features	Progressive brain atrophy in neuroimaging studies, particularly in the cerebellum and brain stem; mild supratentorial atrophy and abnormal gyration	Simplified gyration with normal corpus callosum and brainstem, cerebellar atrophy was reported in one case	2/2 Simplified gyration with cerebral atrophy	4/4 Simplified gyration with cerebral atrophy
Dysmorphic features	Narrow forehead, epicanthic folds, short nose, open mouth, receding chin, and tapered fingers	Not reported	Not reported	4/4 Bitemporal narrowing, puffy cheeks, anteverted nares, tented vermilion of upper lip, short/narrow forehead, high narrow palate
Gingival hypertrophy	Present	Not reported	Not reported	2/4
Ophthalmic manifestations	Absence or early loss of visual fixation with atrophy of optic discs by 2 years of age; normal electroretinogram, extinguished visual evoked potentials	Not reported	Not reported	4/4 Normal
				3/4 Normal
Skeletal manifestations	Poor modeling of long bones, extreme slenderness, and scoliosis osteopenia in all patients	Hypoplastic epiphyses and mild osteopenia	Not reported	4/4 Slender bones with thin cortices, and scoliosis osteopenia
Edema of hands and feet	Present	Reported in one case	Not reported	2/4
Liver enzymes	Not reported	Abnormal in most of the cases	Not reported	Abnormal in some cases
Mutation analysis	NK	EIF2AK3	2/2 IER3IP1	4/4 IER3IP1
Inheritance	AR	AR	AR	AR

NK, not known; AR, autosomal recessive; IGF, insulin-like growth factor; N, number of patients reported.

Epilepsy and EEG changes are rarely associated with Wolcott–Rallison syndrome. In contrast, our patients had early infantile-onset intractable epilepsy with burst suppression EEG pattern characterizing MEDS. Early infantile epileptic encephalopathy with suppression burst (EIEE) is a rare condition known to progress quickly to a vegetative state or death. Despite some progress in understanding the molecular basis of epilepsy, the pathogenesis of EIEE remains unclear [Molinari, 2010]. EIEE is unusual in patients with primary microcephaly and has been reported only in a few patients [Shen et al., 2010; Yu et al., 2010]. Therefore, EIEE and microcephaly appear to be pronounced in MEDS caused by mutations in *IER3IP1*.

All of our patients had dysmorphic facial features similar to those found in patients with progressive encephalopathy, edema, hypsarrhythmia, and optic atrophy (PEHO) syndrome [Somer, 1993] (Table I). These features include a narrow forehead, anteverted nares, prominent uplifted forward-facing ear lobes, puffy cheeks, small chin, characteristic deviation of the eyes, tented vermilion of upper lip, high palate, and gingival hypertrophy. Infantile spasms with hypsarrhythmia, another severe type of infantile-onset epilepsy, is often observed in PEHO syndrome. In addition, patients with PEHO syndrome and the cases reported herein display very similar skeletal changes including scoliosis, kyphosis, and poor molding of long bones that showed extreme slenderness and osteopenia [Somer, 1993]. Despite these overlapping facial and skeletal findings, our patients do not fulfill the clinical diagnostic criteria for PEHO syndrome, and do not display progressive cerebellar atrophy. Furthermore, the presence of congenital microcephaly with simplified gyral pattern, permanent neonatal diabetes, and abnormal liver enzymes also argue against PEHO syndrome diagnosis.

Our results suggest that Wolcott–Rallison syndrome patients and patients with MEDS represent a continuum of phenotypically related neurodevelopmental disorders in which the mutant gene and the nature of the mutation can often predict the severity of the phenotype. Thus, it is possible that inadequate knowledge and the rarity of cases may account, in part, for the confusion in the literature regarding these disorders. Further detailed reports of similar cases along with molecular data hopefully will clarify the questions raised on genotype–phenotype correlation in this group of related disorders.
